# Supplementary material for: Patterns of Intron Gain and Loss in Fungi
Source: PLoS Biol. 2004 Nov 30;2(12):e422. doi: 10.1371/journal.pbio.0020422 (PMC532390; doi:10.1371/journal.pbio.0020422)
Supplement: Table S1 — Also available at http://genes.mit.edu/NielsenEtAl/. (4.3 MB ZIP). [file pbio.0020422.st001.zip › NielsenEtAl/html/795.html]

AN1096.1.NCU05726.1.MG04929.1.FG02730.1


```
 CLUSTAL W (1.82) Multiple Sequence Alignments - Introns Inserted


Sequence 1: MG04929.1	749 aa
Sequence 2: FG02730.1	638 aa
Sequence 3: NCU05726.1	677 aa
Sequence 4: AN1096.1	622 aa
Alignment Length: 785 aa
Number Identitical Residues: 116 aa
Alignment Score (without introns) 6035


MG04929.1 	MYPGASRQLAPLAYQFPPSPIGSSSSIPTRDILQHSKNPSPGGAALLLSGLRTPPSDDMG
NCU05726.1	---------------------------------------------------------MFN
FG02730.1 	----------------------------------------------------------MS
AN1096.1  	---------------------------------------------------------MYP
          	                                                            

MG04929.1 	TTYQAPNIGQHDANPYPSLNGYSGPRSRSSVSSGQAVLDSYPRYHQYATQVTGREHQLPA
NCU05726.1	NSESLCSYSDTQSLYLPNPQQWSTAASSTSSNSGSAQRGYAQQHQQYQQRQHQSRQQSPP
FG02730.1 	TVFQPPLAWESHA-----IHNYAAAVPQAARAP--ATMNEKPQYHRYPVKQP-EQQQQPQ
AN1096.1  	TAQPTLSYLAAPPITPTLLHDSQTDASAFNKQHHQNPLDLPDLSRTAPFRDGLPTPPNDM
          	.           .   .  :      .       .   .     :    :          

MG04929.1 	TQASQQQPSQVA-QPQPIVRESQHTQAT-ATSAVSYPPQAAVAQPSRASTPKSSGTVASQ
NCU05726.1	RQQRQQQYVSQK-QQQQYQQQHQSRKPS-------------SQRPSRPPSP----SPIRR
FG02730.1 	PQTAYNHYYQQE-QATHELHPSQVPQHRNGSISTASQVVPVVGQAVRPSTP-----AAST
AN1096.1  	NGVAYSNIHHYGGKPDPYTGPVYGEGPAYPRMSADLLNRMTRQTQPSQPAQQSSKQAPRE
          	     .:     .:                  :               .: .::      

MG04929.1 	VSMSQKDVASLAGQSLEIPMCISPRGGSLSVLAAQ0MTTFFWFEPCPVHHLAEKLVSSSP
NCU05726.1	EQEISKRDAAMALHSLQVPACISPKGGSLSDLVAD~LTALFWFETSKVLERAATYDALRP
FG02730.1 	STATKNDAESLVYHSLVIPKCISPTGGNLADFAAQ0MTCLFWFESIDELKQAGSVRTMGS
AN1096.1  	RDERPKEKETSKPSYLQIPPSINNGKGSLPDFAAQ0MTCLFWFESTPKLRAVEENS----
          	     :   :     * :* .*.   *.*. :.*: :* :****.    . .        

MG04929.1 	-SEPVPSLNALAIPTPEFKSWVHSVLSTTQVTQNVILLALLFIHRLKTTVPDVRGARGSE
NCU05726.1	GAAPVRRIEGPAIACPNFKKWVHNLLSTTQVTQNVILLSLLYIHRLKVLNPKMHGLPGSE
FG02730.1 	-NAIVQRLPHLAKPLDQFRKWVYSVLSTTQVTQNVILLALLFIYRLKMSTPQIKGRAGSE
AN1096.1  	--AHSLSLCPEAIPTPAFQKWVSNILSTTQVSQNVILLALLFIYRLKKFNPAVRGKKGSE
          	       :   * .   *:.** .:******:******:**:*:***   * ::*  ***

MG04929.1 	YRLLTVALMLGNKF1LDDNTYTNKTWAEVSGITVKDIHVMEVEFLSNMRYSLLVSKAQWE
NCU05726.1	YRLLTVALMLANKF~LDDNTYTNKTWSEVSQLSVNEIHVMEVEFLGNMRYSLLVTEKQWE
FG02730.1 	YRLLTVALMLGNKF1LDDNTYTNKTWAEVSCFNVQEIHVMEVEFLSNMRYNLVATKEQWD
AN1096.1  	YRLMTVALMLGNKF1LDDNTYTNKTWADVSRISVQEIHVMEVEFLSNLRYNLFASEKDWA
          	***:******.*** ***********::** :.*::*********.*:**.*..:: :* 

MG04929.1 	QWLVKLSKFWEYCERATRPVLSPITIPSPTRSVANASIRSPTGHLVLTPTLHSTPTTRSP
NCU05726.1	EWLVKLARFREYLERARQVSSPADLLAPSPNALPVSALPSPTTGQHFPSPLQPTLAGQSP
FG02730.1 	DWLDKLACFHEYYERAVRLPASPVHLPTPTNNTRHSPIASPAGAMQPTVSLPPTPAITAN
AN1096.1  	QWHVKLGRFSDFVNHAPVASENDTLPTPPVRLLSPNSGPTRTQVSPTSSTRLPSPTTEPL
          	:*  **. * :: ::*          ... .     .  : :     . .  .: :  . 

MG04929.1 	YDVTYAANNQWPGQATPQTCSAVSGQLDSRNGTNKRAYSDE-DPTEPPSKR---------
NCU05726.1	SASFYAKQSSGQGWPT-HAAGQTPIWNPASTPLTRKRSMPEFDLLEPPAKRRTPLKPYAS
FG02730.1 	YSPTSSHSQNWSTYQT-NTISPLSTKPSLQFPPVSRKRSPEGDIMEHPAKR---------
AN1096.1  	HPQTWAPVNGSYGTAHQAASQLPPVSS-------RKRRYEEPVEEQHPSKR---------
          	     :  .         :    .           :    *    : *:**         

MG04929.1 	------VAMHPQSIPAQPANMYSQARQRPAASLEPLRLPVPNLTVNTAPHPAQAAYN---
NCU05726.1	SETLYPTVTVPEALRNSREFACAPLPSTSEMALKQARLSVSNLTLNNAQSSTANTLP---
FG02730.1 	------LAPSQR----GPAPVMAQVP-RSNGIVDPARLPVPHPTVMTGQPQTQHTLMPFD
AN1096.1  	--------IVLPHAVPLPSSTLPPASQISAPALPPMMAPTSAPPQQSIPGPVSRLSS---
          	                      .   . .   :     ...  .  .    .        

MG04929.1 	-------SVVSHVPIQQSALSLPPMVPGVRAMATVYPSTTTTSNFSGNVPTSSVPTSVST
NCU05726.1	-----GEVYSRVSYAPESMISLPPILLPAPGMAATYVNSTSAAAPMPSILPTSGSGVSPS
FG02730.1 	NIGANGYPPPTQQVTQPGHVSLPPLQTGMRAMSTVYSSVPAAMVPKQSLPATTGVTMAQT
AN1096.1  	--------SHAFVPSVPASIPQLSAVPGRPTMPPVYNPSTWAPQIPASTAPQPLNNSVPT
          	                 . :.  .       *...*   . :     .  . .      :

MG04929.1 	PLTNSAPGGFPPMTNYATPTKRHSPGSLTPAAAAALGNSSPLSESFGHG----QHGVRTP
NCU05726.1	TVVAAST------TAHPTSQYALSANRLQS------QHSLTSSGAYLGSSPIGEPLIHTP
FG02730.1 	SFPTQAP------VNYGTPSKQLSPGRLGP------FNSSPLAEAYGQSSVIHTPMTHTP
AN1096.1  	PALSLPD-----------PSRHHSPYPVTS------ATISPAVSAYG---------VHTP
          	.    .            .    *.  : .          .   ::           :**

MG04929.1 	ISQSP--SVYLQHRSSPYRPVRHVNTLLCPPPSSSLHEYHLSGPNPTMVAPTQMHYQPLG
NCU05726.1	MSASSPSNIYLQQRTSPYRPVRHAETLLHPPPSAFLQQYHLPNP----IPPPQLHYQPLG
FG02730.1 	ISNSP--SVYLQQRPSPYKPVRHVNTLLHPPPSASLEQYHLSVP----VPPTQMHYQPIG
AN1096.1  	TTHLSP-SYFLANRNSPYRPVRSVNTLLIPPPSASLEQQR-------AIPFHHMHYQPLG
          	 :  .. . :* :* ***:*** .:*** ****: *.: :        :.  ::****:*

MG04929.1 	RRHEYHTGIVPEFTLSAQQQQAQAHYARQEIPMPPTYRQMPGQAQMRPVVGYNLQEPTHS
NCU05726.1	RRNEVRTGIVPEFAMPNSGLTMAG--------AGRYQEPIPAFRQSFSLPLRDIPPATSA
FG02730.1 	RRNDLRTGIVPDFVVYDR--------------VHPQHQALQGHYPS--------------
AN1096.1  	KSTERRTGLLPYLHHDAWPQGP---------YIPPSFHHTPHYAP---------------
          	:  : :**::* :                        .                      

MG04929.1 	HMQFLN-
NCU05726.1	YSQQPRR
FG02730.1 	-------
AN1096.1  	-------
          	
```
